# Do heart failure status and psychosocial variables moderate the relationship between leisure time physical activity and mortality risk among patients with a history of myocardial infarction?

**DOI:** 10.1186/s12872-016-0363-7

**Published:** 2016-10-12

**Authors:** Rony Oosterom-Calo, Saskia J. te Velde, Wim Stut, Yaacov Drory, Johannes Brug, Yariv Gerber

**Affiliations:** 1 Philips Research, Eindhoven, The Netherlands; 2 EMGO Institute for Health and Care Research and the Department of Epidemiology and Biostatistics, VU University Medical Center, Amsterdam, The Netherlands; 3 Department of Rehabilitation, Sackler School of Medicine, Tel Aviv University, Tel Aviv, Israel; 4 Department of Epidemiology and Preventive Medicine, School of Public Health, Sackler Faculty ofMedicine, Tel Aviv University, Tel Aviv, Israel; 5 AmsterdamSchool for Communication Research, University of Amsterdam, Amsterdam, The Netherlands

**Keywords:** Leisure time physical activity, Post-MI, Heart failure, Mortality, Exercise, Psychosocial variables, Depression, Social support

## Abstract

**Background:**

Leisure time physical activity (LTPA) is inversely related to mortality risk among patients with a history of myocardial infarction (MI). The aims were to explore if heart failure (HF) status and psychosocial variables moderate the association.

**Methods:**

Participants (*n* = 1169) were from a multi-center prospective cohort study. Information on LTPA (none, irregular,1–150, 151–300 and >300 weekly minutes), depression, social support and other prognostic indicators were collected 10–13 years after index MI. Cox regressions were conducted, adjusting for potential confounders. In case of significant moderation by HF-status or psychosocial variables, stratified analyses were performed.

**Results:**

During follow-up (M = 8.4 years), 25.6 % of the sample had died. LTPA was inversely associated with mortality (*p* for trend < 0.01 in all models). HF did not, but psychosocial variables did, moderate the association. In the LTPA category 1–150 weekly minutes, patients with a high level of depression had a lower mortality risk in comparison to those with a low level (hazard ratios (95 % confidence intervals) were 0.43 (0.25, 0.75) versus 0.69 (0.36, 1.32)), and patients with a low level of social support had a lower mortality risk in comparison to those with a high level (0.40 (0.21, 0.77) versus 0.71 (0.39, 1.27)). In the category >300 min, patients with a high level of social support had a lower mortality risk than those with a low level (0.38 (0.19, 0.79) versus 0.51 (0.30, 0.87)).

**Conclusions:**

LTPA was inversely related to mortality risk of post-MI patients. HF did not moderate the relationship; depression and social support partially did.

**Electronic supplementary material:**

The online version of this article (doi:10.1186/s12872-016-0363-7) contains supplementary material, which is available to authorized users.

## Background

Physical activity (PA) is recommended to patients that have survived a myocardial infarction (MI) [1]. Physical activity is defined as any bodily movement produced by contraction of skeletal muscles resulting in energy expenditure above the basal level [1]. It may be promoted in a counseling intervention [1, 2] and as part of a cardiac rehabilitation program, including exercise-based training and lifestyle counseling components [3]. Such interventions have shown that performance of PA or exercise can reduce the risk of mortality by follow-up [3, 4]. The association between *leisure time physical activity* (LTPA), including leisurely walking and other recreational and sports activities, on clinical outcomes, also deserves attention. Previous work [5] has demonstrated a strong association between LTPA and mortality among post- MI patients, when comparing performance of no LTPA to performance of weekly regular LTPA and irregular LTPA. Building on the previous work, in the current work we explore differences in mortality risk also based on the number of regular weekly LTPA minutes by refining the category of regular weekly LTPA to include more categories, in order to understand if patients who performed more regular LTPA had a lower mortality risk than those who performed less regular LTPA.

To further explore the benefits of LTPA in post-MI patients, it is worthwhile to uncover which groups of patients may benefit the most from LTPA. Patients at a higher risk of mortality may have a greater need for preventive measures, including performance of LTPA, to reduce the risk. Heart failure (HF) may be an important modifier of the relationship between performance of LTPA and mortality risk, because although on the one had post-MI HF patients have worse prognosis [6] compared to patients who did not develop HF [7], PA performance may be a protective factor among HF patients by reducing the risk of cardiovascular events and mortality [8]. Since post-MI patients who developed HF are at risk of mortality not only due to having had an MI in the past, but also due to their HF, LTPA, as a protective factor may demonstrate a larger effect in terms of reduction of mortality risk. The first aim of the current work is thus to assess whether HF is a modifying factor in the relationship between LTPA and mortality risk.

Also psychosocial factors may have an effect on prognosis [9]. A systematic review and meta-analysis reveals that two years after initial assessment of coronary heart disease (CHD), depressed patients are more than twice as likely to die as non-depressed patients [10] and that socially unsupported post-MI patients have a 2–3-fold higher risk of mortality [11]. Since depressed and socially unsupported patients have a higher risk of mortality than non-depressed and socially supported patients, and therefore more room for improvement in terms of reduction of mortality risk, they may benefit relatively more from LTPA. Specifically, in their case, LTPA may be a protective factor due to having had an MI in the past, as well as due to their psychosocial status, and may therefore demonstrate a larger effect on mortality risk. The second aim is therefore to assess if depression and/or social support moderate the relationship between LTPA and mortality risk among patients with a history of MI.

Finally, a secondary question remains as to whether these high-risk patients actually participate in LTPA. Patients with HF have a reduced exercise tolerance [12], making it difficult for them to exercise. They may therefore be less likely to perform activity than post-MI patients who are HF-free. This could mean that the patients who may benefit the most from LTPA may also be less likely to engage in it, and therefore do not gain the potential benefits. The third aim is therefore to assess the relationship between HF status and LTPA.

**Fig. 1:**
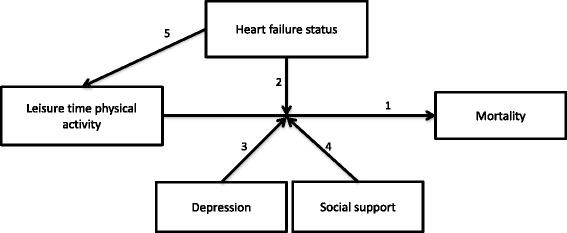
Conceptual model depicting the hypothesized associations between the variables^1^. ^1^Hypothesis 1: Leisure time physical activity will be related to reduced mortality rates; Hypothesis 2: Heart failure status will moderate the relationship between leisure time physical activity and mortality, such that heart failure patients will benefit more than HF-free patients; Hypothesis 3: Depression will moderate the relationship between leisure time physical activity and mortality, such that patients with high scores will benefit more than patients with low depression scores; Hypothesis 4: Social support will moderate the relationship between leisure time physical activity and mortality, such that patients with low scores will benefit more than patients with high social support scores; Hypothesis 5: Patients with HF, high depression scores and/or low social support scores will perform less leisure time physical activity

In summary, we hypothesize that (1) more LTPA is inversely related to mortality risk (Fig. 1), (2) HF status moderates this relationship, such that HF patients benefit relatively more from higher levels of LTPA, (3) depression and (4) social support moderate the relationship between LTPA and mortality risk, such that more depressed and less social supported patients benefit relatively more from higher levels of LTPA, and (5) patients with HF perform less LTPA.

## Method

### Study design and sample

Data were drawn from the Israel Study of First Acute Myocardial Infarction, a longitudinal prospective cohort study investigating the effects of various socio-demographic, medical and psychosocial variables on long-term clinical outcomes and quality of life in patients hospitalized for index MI [13, 14]. The total sample included patients aged 30–65 years, who were admitted to one of eight medical centers in central Israel between February 1992 and February 1993. The parent study was approved by the Ethics Committees of all medical centers involved (Wolfson, Holon; Sheba, Tel Hashomer; Tel Aviv Sourasky, Tel Aviv; Meir, Kfar Sava; Assaf Harofeh, Zerifin; Beilinson, Petach Tikvah; Hasharon, Petach Tikvah; and Laniado, Netanya) and ratified by the Institutional Ethics Committee of Tel Aviv University, and participants gave written informed consent.

### Data collection

Within the Israel Study of First Acute Myocardial Infarction, demographic, socioeconomic and clinical data were collected from medical records and structured interviews one week, 3-6 months, 1–2 years, 5 years and 10–13 years following index MI hospitalization. We used data collected at the latter measurement point, because extensive data on clinical and psychosocial factors were measured in all survivors, in contrast to other measurement points, which targeted a sub-sample, and because it is the most recent measurement point, in which HF is more common (patients are older). Data were obtained through structured interviews, questionnaires, and review of medical records. The data on the following variables was used for the current analysis: Age, sex, educational level, occupational status, HF status, comorbidity status, smoking status, obesity, participation in cardiac rehabilitation, relevant medication prescriptions, depression and social support.

### Variable measures

#### Outcome variable

Follow-up mortality data were obtained through 2011. Death was determined through data from the Israeli Population Registry.

##### Leisure time physical activity

To assess LTPA, patients were asked to rate their LTPA (walking and other activities including gym, exercise at home such as on an exercise bicycle or treadmill, swimming, tennis, soccer/basketball/volleyball, or other) as regular (weekly), irregular (not weekly), or none. Patients that rated their LTPA as regular were asked to report the average frequency and duration of each session [5]. Regular LTPA was divided into three categories: 1–150, 150–300, or >300 weekly minutes. There were therefore five LTPA categories, including no LTPA, irregular LTPA and the three regular LTPA categories.

##### Heart failure status

HF was defined according to clinical history (previous HF-related hospitalization) and a New York Heart Association (NYHA) classification of III-IV.

### Psychosocial variables

Depression was assessed using the depression subscale of the Mental Health Inventory (MHI). The MHI includes 38 items, rated on a 7-point likert scale, ranging from 1 (very strongly disagree) to 7 (very strongly agree). The psychonomic properties of the MHI were assessed on a Israeli sample [15]; the depression subscale had an internal consistency (α) of 0.88. Social support was assessed using the Multidimensional Scale of Perceived Social Support (MSPSS) [16], which has an internal consistency (α) of .92 to .95 for its three subscales [17]. This scale is used to measure the perceived availability of support and includes twelve items, which assess three sources of support: Family, friends, and a significant other. Items are rated on a 7-point likert scale, ranging from 1 (very strongly disagree) to 7 (very strongly agree). The MSPSS has been used in previous research on this dataset [18].

### Confounders

#### Socio-demographic data

Educational level was assessed by asking patients to indicate the number of years of formal education they have received. Occupational status was assessed by asking patients to indicate if they are working, retired, never worked, or stopped working for another reason than retirement.

#### Clinical variables

The clinical variables included conditions other than HF and smoking status. Although there was data available for many conditions within the Israel Study of First Acute Myocardial Infarction, conditions other than HF regarding which information was available (including diabetes, peripheral vascular disease, cerebrovascular disease, cancer, ulcer disease, chronic renal disease, chronic obtrusive pulmonary disease) were selected for inclusion in the current study based on their appearance in the Charlson Comorbidity Index [19]. Conditions were classified dichotomously (having versus not having a condition other than HF). Smoking status, obtained by information from structured interviews [13, 20] was classified as never smoked, previously smoked, or currently smoking. Information on weight and height was used to calculate body mass index (BMI) by dividing the weight by the height squared. Obesity was categorized in three categories, based on the World Health Organization’s (WHO) categorization [21], including below normal until normal weight (BMI < 25, pre-obese (25 < =BMI < 30), and obese (30 < =BMI). Below normal weight and normal weight were grouped in one category due to the small number of below normal weight participants in the sample (*N* = 4).

#### Treatment-related variables

Medication prescriptions were extracted from personal interviews and questionnaires. Patients were asked to indicate if they have ever participated in cardiac rehabilitation.

#### Statistical analyses

Baseline data are presented as percentage or mean (standard deviation). Baseline characteristics across LTPA categories were compared using the using the Mantel–Haenszel chi-square test for trend for categorical variables and generalized linear models for continuous variables. To test the first hypothesis, cox proportional hazards models were constructed to evaluate covariate-adjusted hazards ratios (HR’s) and 95 % confidence intervals (CI’s) associated with LTPA categories. The reference category was defined at no LTPA. Five models, taking into account risk factors for poor post-MI prognosis, were constructed. These models adjusted for: sex and age (basic model, i.e., Model 1); sex, age, educational level and occupational status (demographic model, i.e., Model 2); sex, age, HF status, having a comorbidity, smoking status and obesity (clinical model, i.e., Model 3); sex, age, having received cardiac rehabilitation, being prescribed ACE inhibitors, beta blockers, aspirin, and statins (treatment-related model, i.e., Model 4); and finally, sex, age, depression and social support (psychosocial model, i.e., Model 5). In addition, in order to test the association linearly, the models were tested with LTPA as a continuous variable. The proportional hazards assumption was tested by plotting partial residuals against survival time (i.e. survival, in days, at follow up), with no violations detected.

To test whether the estimated HR’s for the HF and HF-free patients were statistically different from each other, interaction terms between HF-status and each of the LTPA categories were added to the basic model described above. The basic model was selected since it was the most parsimonious of the constructed models and since LTPA was associated with less mortality in all models, including the basic one. A p cutoff value of 0.10 was used as the significance level for the interaction terms, due to difficulties in detecting moderation effects [22], and based on strategies for detecting moderator variables reported elsewhere [23]. In case of significant interaction terms (i.e. *p* < 0.10), HR’s for each group were presented separately. In case of non-significant interaction terms, stratified analyses were only performed to check whether estimated HR’s for each group were indeed similar, or were different but with wide and overlapping confidence intervals.

To test the third and fourth hypotheses, regarding moderation effects of depression and social support, it was tested whether the estimated HR’s for high depression/high social support and low depression/low social support were statistically different from each other by adding interaction terms between depression/social support and each of the LTPA categories to the basic model described above. To explore whether associations between LTPA categories and mortality differed between patients with high and low depression scores, and between socially supported and unsupported patients, stratified analyses were conducted, when significant interaction terms (i.e. *p* < 0.10) were observed, by comparing high to low depression/social groups. Groups were defined using the median scores as a cutoff. Again, in case of non-significant interaction terms, stratified analyses were only performed to check whether estimated hazard ratios for each group were indeed similar, or were different but with wide and overlapping confidence intervals.

To test the fifth hypothesis, the relationship of LTPA with HF status was evaluated using a one-way analysis of variance. Bonferroni post-hoc analyses were performed to test for differences in HF status among LTPA categories. A *p* < 0.05 was regarded as statistically significant. For interaction terms *p* < 0.10 was regarded significant, due to a lower power to detect significant interactions.

## Results

The sample included 1169 patients, who completed the measurement at the fifth interview 10–13 years after index MI (see Additional file 1: Table S1 for sample characteristics). At this time point, 237 (20 %) had developed HF (see Additional file 2: Table S2 for the characteristics of the HF subsample). During a median of 8.4 years follow-up after the fifth interview, 303 deaths occurred (26 % of the sample), of which 122 were in HF cases (i.e., 51 % of the HF cases) and 179 in HF-free cases (i.e., 19 % of the HF-free cases). Of the 303 deaths, 51 % performed no LTPA, 19 % performed irregular LTPA, 13 % performed 1–150 and 8 % performed 151–300 and 8 % performed > 300 weekly minutes of LTPA.

Of the post-MI patients, 37.7 % reported being inactive, 19.1 % reported being irregularly active, 14.5 % reported performing less than 150 weekly﻿ minutes of LTPA, 14.5 % reported performing 151–300 weekly minutes of LTPA, and 13.8 % reported performing more than 300 weekly minutes of LTPA. Men performed more LTPA than women (*p* for trend < 0.01). Educational level and having participated in the past in cardiac rehabilitation were positively related to LTPA (p for trend < 0.01). Having HF, having at least one other comorbidity than HF and depression were negatively related to LTPA (p for trend < 0.01). Out of the inactive participants, 35 % had died by follow-up, followed by 26 % of the irregularly active participants, 17 % of the regularly active participants who had performed 1–150, 14.7 % who performed 151–300, and 22.6 % who performed >300 weekly minutes.

### Is more LTPA related to a reduced mortality risk?

Patients who performed irregular, 1–150, 151–300, or >300 weekly minutes of LTPA all had a lower mortality risk than patients who performed no LTPA (*p* < 0.01 for all; Table 1). In addition to the significant categorical relationships, also linear relationships were found; significant linear trends of LTPA were found in all models (p for trend <0.01 for all).

**Table 1:** Hazard ratios (95 % CI’s) for the relationships between leisure time physical activity categories and survival in the post-MI

	Model 1	Model 2	Model 3	Model 4	Model 5
	HR (95 % CI)	HR (95 % CI)	HR (95 % CI)	HR (95 % CI)	HR (95 % CI)
No LTPA (reference)	1	1	1	1	1
Irregular LTPA	0.66 (0.49, 0.90)	0.66 (0.49, 0.90)	0. 76 (0.55, 1.03)	0.69 (0.51, 0.93)	0.79 (0.55, 1.13)
1–150 min	0.49 (0.34, 0.71)	0.49 (0.34, 0.71)	0.64 (0.44, 0.93)	0.52 (0.36, 0.75)	0.53 (0.34, 0.82)
151–300 min	0.30 (0.19, 0.47)	0.33 (0.21, 0.52)	0.40 (0.25, 0.66)	0.33 (0.21, 0.52)	0.39 (0.24, 0.65)
>300 min	0.39 (0.26, 0.58)	0.39 (0.26, 0.58)	0.50 (0.33, 0.76)	0.41 (0.28, 0.61)	0.49 (0.31, 0.76)
*p* for trend ^a^	<0.01	<0.01	<0.01	<0.01	<0.01

*HR* hazard ratio, *95%CI* 95 % confidence interval, *LTPA* Leisure time physical activity

Model 1: Basic model, adjusted for age and sex; Model 2: demographic model = Model 1 + adjustments for educational level and occupational status; Model 3: clinical model = Model 1 + adjustments for HF status, having a comorbidity, smoking status and obesity; Model 4: treatment-related model = Model 1 + adjustments for having received cardiac rehabilitation, being prescribed ACE inhibitors, beta blockers, aspirin, and statins; Model 5 : psychosocial model = Model 1 + adjustments for depression and social support

^a^Based on Cox regression analyses with LTPA as a continuous variable

### Does heart failure status moderate the relationship between leisure time physical activity and reduced mortality risk?

No interaction of HF by LTPA was found (all interaction terms were *p* > 0.30; see Additional file 3: Table S3). Further exploration by means of analyses stratified by HF status confirmed that the observed estimates for the two groups were very similar.

### Does depression moderate the relationship between leisure time physical activity and survival?

Patients with a high level of depression, who performed 1–150 min of weekly LTPA, had a lower mortality risk than patients with a high level of depression who performed no LTPA (*p* = 0.08; Table 2). Patients with a low level of depression did not have a reduction in mortality risk associated with performance 1–150 min of weekly LTPA. There was no difference in terms of mortality risk between depressed and non-depressed patients who performed irregular, 151–300 or >300 weekly minutes of LTPA (all interaction terms had a *p* > 0.10).

**Table 2:** Hazard ratios (95 % confidence Intervals) in the stratified analysis comparing mortality risk related to leisure time physical activity of patients with high versus low depression and high versus low social support

	High depression^a^	Low depression^a^	p-value interaction term	High social support^a^	Low social support^a^	*p*-value interaction term
	HR ^b^ (95 % CI)	HR ^b^ (95 % CI)		HR ^b^ (95 % CI)	HR ^2^ (95 % CI)	
No LTPA: Reference category	1	1		1	1	
Irregular LTPA	0.80 (0.52, 1.24)	0.63 (3.4, 1.15)	0.63	0.82 (0.47, 1.43)	0.69 (0.44, 1.09)	0.56
1–150 min	0.43 (0.25, 0.75)	0.69 (0.36, 1.32)	0.08	0.71 (0.39, 1.27)	0.40 (0.21, 0.77)	0.09
151–300 min	0.28 (0.13, 0.58)	0.45 (0.23, 0.88)	0.41	0.40 (0.20, 0.80)	0.30 (0.14, 0.62)	0.45
>300 min	0.48 (0.29, 0.81)	0.42 (0.20, 0.86)	0.82	0.38 (0.19, 0.79)	0.51 (0.30, 0.87)	0.10
*p* for trend	>0.01	>0.01	0.39	>0.01	>0.01	0.56

*HR* hazard ratio, *CI* confidence interval, *LTPA* leisure time physical activity

^a^Based on a cutoff value of median depression = 7 and median social support = 5.6

^b^HR adjusting for age and sex

### Does social support moderate the relationship between leisure time physical activity and mortality risk?

The interaction between social support and LTPA was significant for the category 1–150 min (*p* = 0.09; Table 2). Patients with a low level of social support who performed 1–150 min of weekly LTPA had a lower mortality risk compared to patients with a low level of social support who performed no LTPA. Patients who had a high level of social support did not have a reduction in mortality risk associated with performance of 1–150 weekly minutes of LTPA. The interaction was borderline significant for the category >300 min (*p* = 0.10). Patients with high social support who performed >300 min had a lower mortality risk than patients with a high social support who performed no LTPA. A similar relationship was not observed in patients who had a low level of social support. All other interaction terms of LTPA categories by social support were found non-significant (*p* > 0.10).

### Is heart failure status related to performance of leisure time physical activity?

Heart failure status was significantly related to LTPA (p for trend <0.01). Bonferroni post-hoc analyses demonstrated that patients who performed no LTPA were more likely to have HF than those in all other LTPA categories (*p* < 0.01). Patients who performed irregular LTPA were more likely to have HF in comparison to those that performed 151–300 weekly minutes of LTPA (*p* = 0.04). Differences between all other LTPA categories were non-significant.

## Discussion

A persistent, inverse association between LTPA and mortality risk was demonstrated in the current study in a sample of post-MI patients, irrespective of HF status and when adjusting for basic, demographic, clinical, treatment-related and psychosocial covariates. These results replicate previous work demonstrating the survival benefits related to being active for post-MI patients [5]. The current work adds to previous investigations through the refinement of the regular weekly LTPA variable into three categories based on minutes of LTPA per week, and by exploring potential moderators for the association, thereby allowing making practical recommendations for LTPA promotion. Moreover, previous studies with HF patients have focused on exercise training [24, 25] and on PA following a counseling intervention [4]. This is the first study to focus specifically on PA conducted at leisure time among HF patients.

Our hypothesis regarding the moderating effects of HF status on the inverse relationship between LTPA and mortality risk was not met. This indicates that HF patients were just as likely to benefit from LTPA as HF-free patients with a history of MI. Therefore, interventions to promote LTPA may be beneficial for inactive patients with and without HF. It may be necessary, however, to target interventions separately at the two groups because HF and HF free post-MI patients differ from each other on a number of characteristics, including age [7].

Depression and social support partially moderated the relationship between LTPA and mortality risk. It was found that patients with high depression scores, as well as those with low social support scores, appear to benefit from *any* amount of regular, weekly, LTPA, i.e. that low levels of LTPA are already beneficial for these groups. It was also found that performing more than 300 min of LTPA was more strongly related to survival in the high social support group than in the low social support group, although both groups benefitted from conducting more than 300 min of LTPA.

Finally, it was found that patients with HF were less likely to perform LTPA in comparison to those without HF. This indicates that in particular HF patients may have a need for LTPA promotion interventions, especially in light of the fact that they benefit from LTPA just as much as the HF-free patients. There is evidence that preventive interventions targeting specifically the HF patient population, can be effective [4]. More research is necessary to clarify the direction of causality, however, as there is also evidence that patients who are less active are more likely to develop HF [26].

There are a number of strengths in the current work. The results make an important contribution to current knowledge about the relationship among LTPA and mortality risk among post-MI patients. It replicates results regarding the survival benefits of PA performed at leisure among patients with a history of MI, but using a more refined LTPA variable, thereby contributing to more specific recommendations regarding LTPA promotion. It is the first study exploring the moderating effects of HF status on this relationship. Since prognosis is low among HF patients [6] this association is of particular importance. The inverse association between LTPA performance and mortality risk was evaluated in models that adjusted for a range of relevant variables, thereby increasing the confidence in the observed effects.

There are also a number of limitations. Based on the results, it is unclear if certain characteristics of active patients, for example a better health status, can account for the observed associations between LTPA and survival. Known mortality risk factors were adjusted for in the statistical models, but it is still possible that factors beyond the scope of the current work account for the findings. A limiting factor of the current work is that the baseline mortality risk of participants was not taken into account. However, with survival analyses it is not possible to estimate the baseline risk. Performing another type of analysis, such as logistic regression, would not have been preferable in this case, because it would have not been possible to take time into account in the model. Selection bias may have also been a limitation. The selection of HF patients was based on hospitalization history and a NYHA III-IV class, rather than the Framingham criteria for HF diagnosis [27]. Furthermore, patients who died before index MI, as well as patients who died during index hospitalization, were not included. Finally, LTPA measurement was not optimal, because it was self-reported, rather than assessed with objective measurement techniques such as accelerometers [28]. It is often difficult for economic and pragmatic reasons to assess LTPA with accelerometers in prospective cohort studies, but some studies have done this [29–31], although notably with smaller sample sizes. Furthermore, the self-report measure of LTPA provided information regarding duration of LTPA but not on exercise intensity or about more specific modes of physical activity, and effects of LTPA may be dependent on intensity and may be different for specific activities.

## Conclusions

In conclusion, in a sample of post-MI Israeli patients, LTPA positively predicted reductions in mortality risk, irrespective of HF status, although HF patients were less likely to be physically active than HF-free patients. There was some evidence that depression and social support moderate the inverse relationship between LTPA and mortality risk, indicating that more depressed patients and patients with less social support may already benefit from relatively fewer weekly minutes of LTPA. These findings have implications for LTPA promotion among patients with a history of MI.

## Additional files

Additional file 1: Table S1. Baseline characteristics of the post-MI patient sample. Baseline characteristics of the post-MI sample categorized according to amount of LTPA performed. (DOC 61 kb)
Additional file 2: Table S2. Baseline characteristics of the HF subsample. Baseline characteristics of the subsample of post-MI patients who developed HF categorized according to amount of LTPA performed. (DOC 58 kb)
Additional file 3: Table S3. Hazard ratio’s (CI’s) in the stratified analysis comparing HF to HF-free cases’ mortality risk related to LTPA categories. Results of the stratified analysis comparing the mortality risk in relation to LTPA of post-MI patients who did and did not develop HF. (DOC 41 kb)

## Abbreviations

BMI: Body mass index
CI: Confidence interval
HF: Heart failure
HR: Hazards ratio
LTPA: Leisure time physical activity
MI: Myocardial infarction
WHO: World health organization

## References

[1] Corra U, Piepoli MF, Carre F, Heuschmann P, Hoffmann U (2010). Secondary prevention through cardiac rehabilitation: physical activity counselling and exercise training: key components of the position paper from the Cardiac Rehabilitation Section of the European Association of Cardiovascular Prevention and Rehabilitation. Eur Heart J.

[2] Piepoli MF, Guazzi M, Boriani G, Cicoira M, Corra U (2010). Exercise intolerance in chronic heart failure: mechanisms and therapies. Part I. Eur J Cardiovasc Prev Rehabil.

[3] Giannuzzi P, Temporelli PL, Marchioli R, Maggioni AP, Balestroni G (2008). Global secondary prevention strategies to limit event recurrence after myocardial infarction: results of the GOSPEL study, a multicenter, randomized controlled trial from the Italian Cardiac Rehabilitation Network. Arch Intern Med.

[4] Wal M, Veldhuisen D, Veeger N, Rutten F, Jaarsma T (2010). Compliance with non-pharmacological recommendations and outcome in heart failure patients. Eur Heart J.

[5] Gerber Y, Myers V, Goldbourt U, Benyamini Y, Scheinowitz M (2011). Long-term trajectory of leisure time physical activity and survival after first myocardial infarction: a population-based cohort study. Eur J Epidemiol.

[6] Mosterd A, Cost B, Hoes AW, de Bruijne MC, Deckers JW (2001). The prognosis of heart failure in the general population: The Rotterdam Study. Eur Heart J.

[7] Emanuelsson H, Karlson BW, Herlitz J (1994). Characteristics and prognosis of patients with acute myocardial infarction in relation to occurrence of congestive heart failure. Eur Heart J.

[8] Miura Y, Fukumoto Y, Miura T, Shimada K, Asakura M (2012). Impact of physical activity on cardiovascular events in patients with chronic heart failure. A multicenter prospective cohort study. Circ J.

[9] Hemingway H, Marmot M (1999). Evidence based cardiology: psychosocial factors in the aetiology and prognosis of coronary heart disease. Systematic review of prospective cohort studies. BMJ.

[10] Barth J, Schumacher M, Herrmann-Lingen C (2004). Depression as a risk factor for mortality in patients with coronary heart disease: a meta-analysis. Psychosom Med.

[11] Mookadam F, Arthur HM (2004). Social support and its relationship to morbidity and mortality after acute myocardial infarction: systematic overview. Arch Intern Med.

[12] McMurray J, Adamopoulos S, Anker S, Auricchio A, Bohm M (2012). ESC guidelines for the diagnosis and treatment of acute and chronic heart failure 2012: The Task Force for the Diagnosis and Treatment of Acute and Chronic Heart Failure 2012 of the European Society of Cardiology. Developed in collaboration with the Heart Failure Association (HFA) of the ESC. Eur J Heart Fail.

[13] Gerber Y, Rosen LJ, Goldbourt U, Benyamini Y, Drory Y (2009). Smoking status and long-term survival after first acute myocardial infarction a population-based cohort study. J Am Coll Cardiol.

[14] Gerber Y, Benyamini Y, Goldbourt U, Drory Y (2010). Neighborhood socioeconomic context and long-term survival after myocardial infarction. Circulation.

[15] Florian V, Drory Y (1990). The mental health inventory: psychometric properties and normative data in the Israeli population. Psychologia.

[16] Zimet GD, Powell SS, Farley GK, Werkman S, Berkoff KA (1990). Psychometric characteristics of the multidimensional scale of perceived social support. J Pers Assess.

[17] Zimet GD, Dahlem NW, Zimet SG, Farley GK (1988). The multidimensional scale of perceived social support. J Pers Assess.

[18] Drory Y, Kravetz S, Hirschberger G (2002). Long-term mental health of men after a first acute myocardial infarction. Arch Phys Med Rehabil.

[19] Charlson ME, Pompei P, Ales KL, MacKenzie CR (1987). A new method of classifying prognostic comorbidity in longitudinal studies: development and validation. J Chronic Dis.

[20] Gerber Y, Koren-Morag N, Myers V, Benyamini Y, Goldbourt U (2011). Long-term predictors of smoking cessation in a cohort of myocardial infarction survivors: a longitudinal study. Eur J Cardiovasc Prev Rehabil.

[21] Organization WH (1997). Preventing and managing the global epidemic [report of a WHO Consultation on Obesity].

[22] McClelland GH, Judd CM (1993). Statistical difficulties of detecting interactions and moderator effects. Psychol Bull.

[23] Stone-Romero EF, Liakhovitski D (2002). Strategies for detecting moderator variables: a review of conceptual and empirical issues. Research in personnel and human resources management.

[24] Belardinelli R, Georgiou D, Cianci G, Purcaro A (1999). Randomized, controlled trial of long-term moderate exercise training in chronic heart failure: effects on functional capacity, quality of life, and clinical outcome. Circulation.

[25] O’Connor CM, Whellan DJ, Lee KL, Keteyian SJ, Cooper LS (2009). Efficacy and safety of exercise training in patients with chronic heart failure: HF-ACTION randomized controlled trial. JAMA.

[26] He J, Ogden LG, Bazzano LA, Vupputuri S, Loria C (2001). Risk factors for congestive heart failure in US men and women: NHANES I epidemiologic follow-up study. Arch Intern Med.

[27] McKee PA, Castelli WP, McNamara PM, Kannel WB (1971). The natural history of congestive heart failure: the Framingham study. N Engl J Med.

[28] Prince S, Adamo K, Hamel M, Hardt J, Gorber S (2008). A comparison of direct versus self-report measures for assessing physical activity in adults: a systematic review. Int J Behav Nutr Phys Act.

[29] Tucker JM, Tucker LA, Lecheminant J, Bailey B. Obesity increases risk of declining physical activity over time in women: A prospective cohort study. Obesity (Silver Spring). 2013;21(12):E715–20. 10.1002/oby.20415 23512799

[30] Hamer M, Kivimaki M, Steptoe A (2012). Longitudinal patterns in physical activity and sedentary behaviour from mid-life to early old age: a substudy of the Whitehall II cohort. J Epidemiol Community Health.

[31] van den Berg-Emons RJ, Bussmann JB, Haisma JA, Sluis TA, van der Woude LH (2008). A prospective study on physical activity levels after spinal cord injury during inpatient rehabilitation and the year after discharge. Arch Phys Med Rehabil.

