# Supplementary material for: Do heart failure status and psychosocial variables moderate the relationship between leisure time physical activity and mortality risk among patients with a history of myocardial infarction?
Source: BMC Cardiovasc Disord. 2016 Oct 12;16:196. doi: 10.1186/s12872-016-0363-7 (PMC5059913; doi:10.1186/s12872-016-0363-7)
Supplement: Additional file 1: Table S1. — Baseline characteristics of the post-MI patient sample. Baseline characteristics of the post-MI sample categorized according to amount of LTPA performed. (DOC 61 kb) [file 12872_2016_363_MOESM1_ESM.doc]

Table S1: Baseline characteristics of the post-MI patient sample

|  | **Missing values** | **Total sample** | **No LTPA** | **Irregular** | **<150 minutes LTPA** | **151-300 minutes LTPA** | **>300 minutes LTPA** | ***p* for trend ^1^** |
| --- | --- | --- | --- | --- | --- | --- | --- | --- |
| **N (%)** | 2 (0.001) | 1169 | 441 (37.7) | 224 (19.1) | 170 (14.5) | 170 (14.5) | 162 (13.8) |  |
| **Age (SD)** | 0 | 63.9 (8.2) | 64.0 (8.7) | 63.4 (7.8) | 63.3 (7.9) | 63.6 (8.0) | 65.5 (7.8) | 0.26 |
| **Sex**  ***Men (%)*** | 0 | 966 (82.6) | 336 (76.2) | 196 (87.5) | 147 (86.5) | 141 (82.9) | 144 (88.9) | 0.00 |
| **Educational level**  ***Mean number of Years (SD)*** | 0 | 11.3 (4.0) | 10.2 (4.1) | 11.6 (4.0) | 12.2 (3.6) | 12.5 (3.7) | 11.7 (3.8) | 0.00 |
| **Occupational status**  ***Working (%)***  ***Not working- Pension/Never worked (%)***  ***Not working- another reason (%)*** | 0 | 508 (43.5)  214 (18.3)  447 (38.2) | 178 (40.4)  76 (17.2)  187 (42.4) | 102 (45.5)  38 (16.9)  84 (37.5) | 70 (41.2)  29 (17.1)  71 (41.8) | 96 (56.5)  30 (17.6)  44 (25.9) | 60 (37.0)  41 (25.3)  61 (37.7) | 0.67 |
| **HF status**  ***Have HF* (%)** | 5 (2.1) | 237 (20.3) | 136 (30.8) | 41 (18.3) | 26 (15.3) | 12 (7.1) | 22 (13.6) | 0.00 |
| **Comorbidity**  ***At least one (%)*** | 0 | 663 (56.7) | 297 (67.3) | 127 (56.7) | 76 (44.7) | 82 (48.2) | 80 (49.4) | 0.00 |
| **Smoking status**  ***Smokers (%)***  ***Never Smoked (%)***  ***Quit (%)*** | 0 | 251 (21.5)  391 (33.4)  527 (45.1) | 121 (27.4)  131 (29.7)  189 (42.9) | 41 (18.3)  82 (36.6)  101 (45.1) | 36 (21.2)  66 (38.8)  68 (40.0) | 25 (14.7)  55 (32.4)  90 (52.9) | 28 (17.3)  56 (34.6)  78 (48.1) | 0.72 |
| **Obesity**  ***Below normal to***  ***normal weight***  ***Pre-obese***  ***Obese*** | 21 | 350 (30)  532 (45)  266 (23) | 126 (29)  180 (42)  123 (29) | 62 (28)  97 (44)  61 (28) | 47 (27)  90 (53)  32 (19) | 56 (33)  79 (47)  32 (19) | 58 (36)  85 (53)  18 (11) | 0.00 |
| **Participated in cardiac rehabilitation *(%)*** | 0 | 224 (19.2) | 52 (11.8) | 40 (17.9) | 50 (29.4) | 44 (25.9) | 37 (22.8) | 0.00 |
| **Taking Aspirin (%)** | 0 | 1007 (86.1) | 364 (82.5) | 200 (89.3) | 150 (88.2) | 149 (87.6) | 142 (87.7) | 0.06 |
| **Taking Statins (%)** | 0 | 821 (70.2) | 294 (66.7) | 156 (69.6) | 125 (73.5) | 133 (78.2) | 112 (69.1) | 0.059 |
| **Taking Beta Blockers (%)** | 0 | 690 (59.0) | 257 (58.3) | 130 (58.0) | 108 (63.5) | 104 (61.2) | 90 (55.6) | 0.95 |
| **Taking ACE inhibitors (%)** | 0 | 554 (47.4) | 208 (47.2) | 108 (48.2) | 87 (51.2) | 76 (44.7) | 75 (46.3) | 0.76 |
| **Depression**  ***MHI depression subscale score (SD)*** | 185 (15) | 8.2 (4.5) | 9.7 (5.3) | 7.7 (4.0) | 7.7 (3.8) | 6.8 (3.5) | 7.4 (3.7) | 0.00 |
| **Social support**  ***MSPSS score (SD)*** | 218 (18) | 5.4 (1.3) | 5.2 (1.4) | 7.0 (5.4) | 5.6 (1.3) | 5.7 (1.1) | 5.5 (1.3) | 0.15 |

^1^ Based on Mantel–Haenszel chi-square test for trend for categorical variables and generalized linear models for continuous variables
