# Supplementary material for: Do heart failure status and psychosocial variables moderate the relationship between leisure time physical activity and mortality risk among patients with a history of myocardial infarction?
Source: BMC Cardiovasc Disord. 2016 Oct 12;16:196. doi: 10.1186/s12872-016-0363-7 (PMC5059913; doi:10.1186/s12872-016-0363-7)
Supplement: Additional file 2: Table S2. — Baseline characteristics of the HF subsample. Baseline characteristics of the subsample of post-MI patients who developed HF categorized according to amount of LTPA performed. (DOC 58 kb) [file 12872_2016_363_MOESM2_ESM.doc]

Table S2: Baseline characteristics of the HF subsample

|  | **Missing values** | **Total sample** | **No LTPA** | **Irregular** | **<150 minutes LTPA** | **151-300 minutes LTPA** | **>300 minutes LTPA** | ***p* for trend** |
| --- | --- | --- | --- | --- | --- | --- | --- | --- |
| **N (%)** |  | 237 | 136 (57.4) | 41 (17.3) | 26 (11.0) | 12 (5.1) | 22 (9.3) |  |
| **Age (SD)** |  | 66.37 (7.3) | 66.5 (7.8) | 66.3 (6.6) | 66.0 (7.3) | 65.4 (5.7) | 66.5 (6.4) | 0.77 |
| **Sex**  ***Men (%)*** | 0 | 179 | 93 (68.4) | 33 (80.5) | 22 (84.6) | 12 (100) | 19 (86.4) | 0.004 |
| **Educational level**  ***Mean number of Years (SD)*** |  | 10.1 (4.5) | 8.99 (4.1) | 10.6 (5.2) | 12.3 (3.4) | 13.7 (14.7) | 11.4 (4.1) | 0.62 |
| **Occupational status**  ***Working (%)***  ***Not working- Pension/Never worked (%)***  ***Not working- another reason (%)*** | 0 | 53 (22.3)  54 (22.8)  130 (54.9) | 24 (17.6)  31 (22.8)  81 (59.6) | 8 (19.5)  9 (22.0)  24 (58.5) | 8 (30.8)  5 (19.2.0)  13 (50.0) | 7 (58.3)  2 (16.7)  3 (25.0) | 6 (27.3)  7 (31.8)  9 (40.9) | 0.21 |
| **Comorbidity**  ***At least one (%)*** | 0 | 200 (84.4) | 22 (89.7) | 35 (85.4) | 20 (76.9) | (66.7) | 15 (68.2) | 0.001 |
| **Smoking status**  ***Smokers (%)***  ***Never Smoked (%)***  ***Quit (%)*** | 0 | 42 (17.7)  79 (33.0)  116 (48.9) | 27 (19.9)  42 (30.9)  67 (49.3) | 5 (12.2)  13 (31.7)  23 (56.1) | 8 (30.8)  11 (42.3)  7 (26.9) | 0 (0.0)  5 (41.7)  7 (58.3) | 2 (9.1)  8 (36.4)  12 (54.5) | 0.58 |
| **Obesity**  ***Below normal to***  ***normal weight***  ***Pre-obese***  ***Obese*** | 6 (2) | 72 (31)  98 (42)  61 (26) | 42 (32)  51(39)  39 (29) | 10 (25)  17 (42)  13 (33) | 9 (36)  10 (40)  6 (24) | 4 (33)  6 (50)  2 (16) | 7 (33)  14 (66)  1(0.5) | 0.18 |
| **Participated in cardiac rehabilitation *(%)*** | 0 | 38 (16.0) | 14 (10.3) | 6 (14.6) | 8 (30.8) | 4 (33.3) | 6 (27.3) | 0.00 |
|  |  |  |  |  |  |  |  |  |
| **Taking Aspirin (%)** | 0 | 201 (84.8) | 113 (83.1) | 34 (82.9) | 24 (92.3) | 10 (83.3) | 20 (90.9) | 0.27 |
| **Taking Statins (%)** | 0 | 164 (69.2) | 89 (65.4) | 23 (56.1) | 23 (88.5) | 11 (91.7) | 18 (81.8) | 0.01 |
| **Taking Beta Blockers (%)** | 0 | 153 (64.6) | 84 (61.8) | 28 (68.3) | 17 (65.4) | 7 (58.3) | 17 (77.3) | 0.25 |
| **Taking ACE inhibitors (%)** | 0 | 138 (58.2) | 72 (52.9) | 24 (58.5) | 17 (65.4) | 9 (75.0) | 16 (72.7) | 0.02 |
| **Depression**  ***MHI depression subscale score (SD)*** | 56 (23.6) | 10.1 (5.5) | 11.6 (6.0) | 9.5 (4.9) | 8.3 (4.2) | 6.1 (1.8) | 8.1 (4.4) | 0.44 |
| **Social support**  ***MSPSS score (SD)*** | 28 (24.4) | 5.1 (1.4) | 4.8 (1.4) | 5.0 (1.2) | 5.8 (0.9) | 5.6 (1.1) | 5.7 (3.3) | 0.12 |

^1^ Based on Mantel–Haenszel chi-square test for trend for categorical variables and generalized linear models for continuous variables
