# Supplementary material for: Do heart failure status and psychosocial variables moderate the relationship between leisure time physical activity and mortality risk among patients with a history of myocardial infarction?
Source: BMC Cardiovasc Disord. 2016 Oct 12;16:196. doi: 10.1186/s12872-016-0363-7 (PMC5059913; doi:10.1186/s12872-016-0363-7)
Supplement: Additional file 3: Table S3. — Hazard ratio’s (CI’s) in the stratified analysis comparing HF to HF-free cases’ mortality risk related to LTPA categories. Results of the stratified analysis comparing the mortality risk in relation to LTPA of post-MI patients who did and did not develop HF. (DOC 41 kb) [file 12872_2016_363_MOESM3_ESM.doc]

Table S3: Hazard ratio’s (CI’s) in the stratified analysis comparing HF to HF-free cases’ mortality risk related to leisure time physical activity categories

|  | Patients with HF | HF-free patients |  |
| --- | --- | --- | --- |
|  | HR ^1^ (95%CI) | HR ^1^ (95%CI) | p-value interaction term^3^ |
| No LTPA: Reference category | 1 | 1 |  |
| Irregular LTPA | 0.70 (0.43, 1.14) | 0.75 (0.50, 1.11) | 0.76 |
| 1-150 minutes | **0.54 (0.20, 1.03)** | **0.59 (0.37, 0.94)** | **0.73** |
| 151-300 minutes | **0.20 (0.04, 0.81)** | **0.42 (0.26, 0.70)** | **0.29** |
| >300 minutes | **0.43 (0.20, 0.90)** | **0.47 (0.29, 0.76)** | **0.67** |
| *p* for trend ^2^ | **>0.01** | **>0.01** | **0.41** |

HR = hazard ratio

95%CI = 95% confidence interval

LTPA = Leisure Time Physical Activity

^1^ HRs adjusted for age and sex

^2^ Based on Cox regression analyses with LTPA as a continuous variable

^3^ Derived from Cox regression analyses including interaction terms of HF-status with each LTPA category
